# Potential inconsistencies in Zika surveillance data and our understanding of risk during pregnancy

**DOI:** 10.1371/journal.pntd.0006991

**Published:** 2018-12-10

**Authors:** James A. Hay, Pierre Nouvellet, Christl A. Donnelly, Steven Riley

**Affiliations:** 1 MRC Centre for Global Infectious Disease Analysis, Department of Infectious Disease Epidemiology, Imperial College, London, UK; 2 School of Life Sciences, University of Sussex, Brighton, UK; 3 Department of Statistics, University of Oxford, Oxford, UK; University of Pittsburgh, UNITED STATES

## Abstract

**Background:**

A significant increase in microcephaly incidence was reported in Northeast Brazil at the end of 2015, which has since been attributed to an epidemic of Zika virus (ZIKV) infections earlier that year. Further incidence of congenital Zika syndrome (CZS) was expected following waves of ZIKV infection throughout Latin America; however, only modest increases in microcephaly and CZS incidence have since been observed. The quantitative relationship between ZIKV infection, gestational age and congenital outcome remains poorly understood.

**Methodology/Principle findings:**

We characterised the gestational-age-varying risk of microcephaly given ZIKV infection using publicly available incidence data from multiple locations in Brazil and Colombia. We found that the relative timings and shapes of ZIKV infection and microcephaly incidence curves suggested different gestational risk profiles for different locations, varying in both the duration and magnitude of gestational risk. Data from Northeast Brazil suggested a narrow window of risk during the first trimester, whereas data from Colombia suggested persistent risk throughout pregnancy. We then used the model to estimate which combination of behavioural and reporting changes would have been sufficient to explain the absence of a second microcephaly incidence wave in Bahia, Brazil; a population for which we had two years of data. We found that a 18.9-fold increase in ZIKV infection reporting rate was consistent with observed patterns.

**Conclusions:**

Our study illustrates how surveillance data may be used in principle to answer key questions in the absence of directed epidemiological studies. However, in this case, we suggest that currently available surveillance data are insufficient to accurately estimate the gestational-age-varying risk of microcephaly from ZIKV infection. The methods used here may be of use in future outbreaks and may help to inform improved surveillance and interpretation in countries yet to experience an outbreak of ZIKV infection.

## Author summary

Zika virus (ZIKV) infection is associated with the rise of microcephaly cases observed in Northeast Brazil at the end of 2015. For women in endemic or at-risk areas, understanding how the relationship between time of infection and microcephaly risk varies through pregnancy is important in informing family planning. However, a relatively modest number of congenital Zika syndrome cases have been observed following subsequent waves of ZIKV infection, limiting our understanding of gestational risk. We used a mathematical model to quantify the shape and magnitude of the gestational-age-varying risk to a fetus. Although the risk profile should be conserved regardless of location, we estimated different profiles when using surveillance data from locations in Northeast Brazil and Colombia. Our results suggest that time-dependent reporting changes likely confound the interpretation of currently available surveillance data. Furthermore, we investigated a range of behavioural and reporting rate changes that could explain two waves of ZIKV infection in Bahia, Brazil despite only one wave of microcephaly. Plausible changes in reporting could explain these data whilst remaining consistent with the hypothesis that ZIKV infection carries a significant risk of microcephaly. Further evidence is needed to disentangle the true risk of congenital Zika syndrome from time-varying reporting changes.

## Introduction

A substantial body of experimental and clinical evidence implicates Zika virus (ZIKV) infection in the sharp rise in the incidence of microcephaly cases in Brazil at the end of 2015. [1–5] Previous population-level studies investigating the relationship between ZIKV and microcephaly incidence found consistent patterns of high first-trimester risk and lower risk later in pregnancy, which is consistent with early clinical findings for ZIKV-associated microcephaly. [6, 7] However, clinical studies investigating the link between ZIKV infection and a distinctive pattern of congenital abnormalities, collectively termed congenital Zika syndrome (CZS), suggest that adverse outcomes are associated with ZIKV infection throughout pregnancy. [8–10] How these clinical findings link to the complex picture portrayed by the epidemiological data in Brazil is still unclear and leaves a substantial knowledge gap for those counseling pregnant women in ZIKV-affected populations. For example, why did the majority of Latin America demonstrate a relatively small rise in microcephaly incidence rates compared to those seen in Northeast Brazil, and why was the second wave of microcephaly in Brazil much smaller than the first despite two similar waves of Guillain-Barré Syndrome (GBS)? [11]

It is useful to consider the conceptual model in which observed population-level CZS incidence reflects underlying ZIKV transmission dynamics and a gestational-age-varying risk of CZS given infection with ZIKV. If a pregnant woman is infected at some point during gestation, her baby may present as a case of CZS with a probability conditional on the gestational age of her baby when infection occurred. Characterizing this link between underlying gestational risk of CZS and its presentation in epidemiological data has two potential benefits. First, an estimate of the underlying gestational risk profile from surveillance data may provide evidence to inform women of childbearing age to help plan pregnancy and mitigate exposure risk. [12] Second, if the risk profiles differ substantially between populations, those differences could support the study of alternative hypotheses of risk factors for CZS beyond ZIKV infection. For example, prior infection with another arbovirus has been suggested as a potential cofactor for risk of GBS, however prior arbovirus infection has not yet been shown to play a role in increased neurological adverse event risk. [13]

Research on other teratogenic pathogens shows the potential importance of gestational age to CZS risk. [14] For example, prospective cohort studies of pregnant women have shown that infection early in gestation greatly increases the risk of congenital rubella syndrome and cytomegalovirus-associated adverse fetal outcomes relative to infection later in pregnancy. [15, 16] Although a similar pattern seems likely to be the case for CZS, the timing and magnitude of risk throughout pregnancy remains uncertain. However, quantifying this underlying gestational-age-varying risk profile should be possible given reliable data on infection and CZS incidence combined and a robust statistical approach. Here, we demonstrate how a transmission model fitted to reported incidence data can be used to infer the relationship between gestational age at the time of ZIKV infection and the risk of microcephaly. We adapt this model to explore potential explanations, including changes in reporting rates and abortions, for the lack of a second observed wave of microcephaly incidence in Brazil.

## Materials and methods

### ZIKV and microcephaly incidence data

We searched the literature, Pan American Health Organization (PAHO), World Health Organization (WHO) and Brazilian state health authority websites for reports of suspected or confirmed ZIKV infection incidence and microcephaly cases in 2015 and early 2016, building on a comprehensive literature search performed in 2016. [17] In particular, we searched www.paho.org, www.who.int, Brazilian state-level ministry of health websites (eg. www.suvisa.ba.gov.br), and PubMed for the terms “zika” and “microcephaly”. Where confirmation status of cases was not recorded (eg. where incidence was only shown as “reported cases”), we classified these data as “notified” cases. Where suspected and confirmed cases were distinguished, we classified the sum of suspected and confirmed cases as “notified” cases. A summary of data included in the analyses can be found in S1 Table.

Coverage, case definitions and protocols for ZIKV and microcephaly surveillance in Brazil and Colombia changed throughout 2015 and 2016, making direct comparison of incidence data between these years difficult. [18] Although the first outbreak of laboratory confirmed ZIKV was reported in Brazil on 07/05/2015, ZIKV reporting only became compulsory through the national notifiable information system (SINAN) on 17/02/2016. [19, 20] Furthermore, laboratory confirmation was only performed on suspected cases from previously unaffected areas and on certain subpopulations of interest (eg. pregnant women, hospitalised patients with neurological complications). Reporting of microcephaly and other congenital abnormalities was through the information on live births system (SINASC) until November 2015, when the new public health events registry (RESP) was implemented to improve surveillance in pregnant women and newborns. [21] In Colombia, passive reporting of ZIKV cases and major congenital abnormalities (including microcephaly) is ongoing through the National Health Institute (INS) surveillance system. [22, 23] Laboratory testing and mandatory reporting of ZIKV infections based on clinical symptoms began on 14/10/2015. [24] However, RT-PCR confirmation was not systematic and, like Brazil, was only used to confirm the presence of ZIKV in municipalities that had not yet detected the virus and in subpopulations at particular risk of complications. Therefore, the incidence of confirmed ZIKV infections gives an indication of the spread of the virus, but not necessarily the true magnitude and dynamics of the epidemic within affected locations.

Some data sources were only available in graphical form, and these numbers were therefore extracted using a web digitiser (https://automeris.io/WebPlotDigitizer/). The results presented in the main text used data from: Northeast Brazil; Colombia; the city of Salvador, Bahia, Brazil; and state-reported incidence from Bahia, Rio Grande do Norte and Pernambuco, Brazil. Numbers of live births were obtained for Brazil from the SINASC/CGIAE/SVS/MS system. [7, 25] For Colombia, live births were obtained from a publication of microcephaly and ZIKV infection incidence in Colombia and from Colombian ministry of health vital statistics. [23, 26]

### Model description

**Fig 1:**
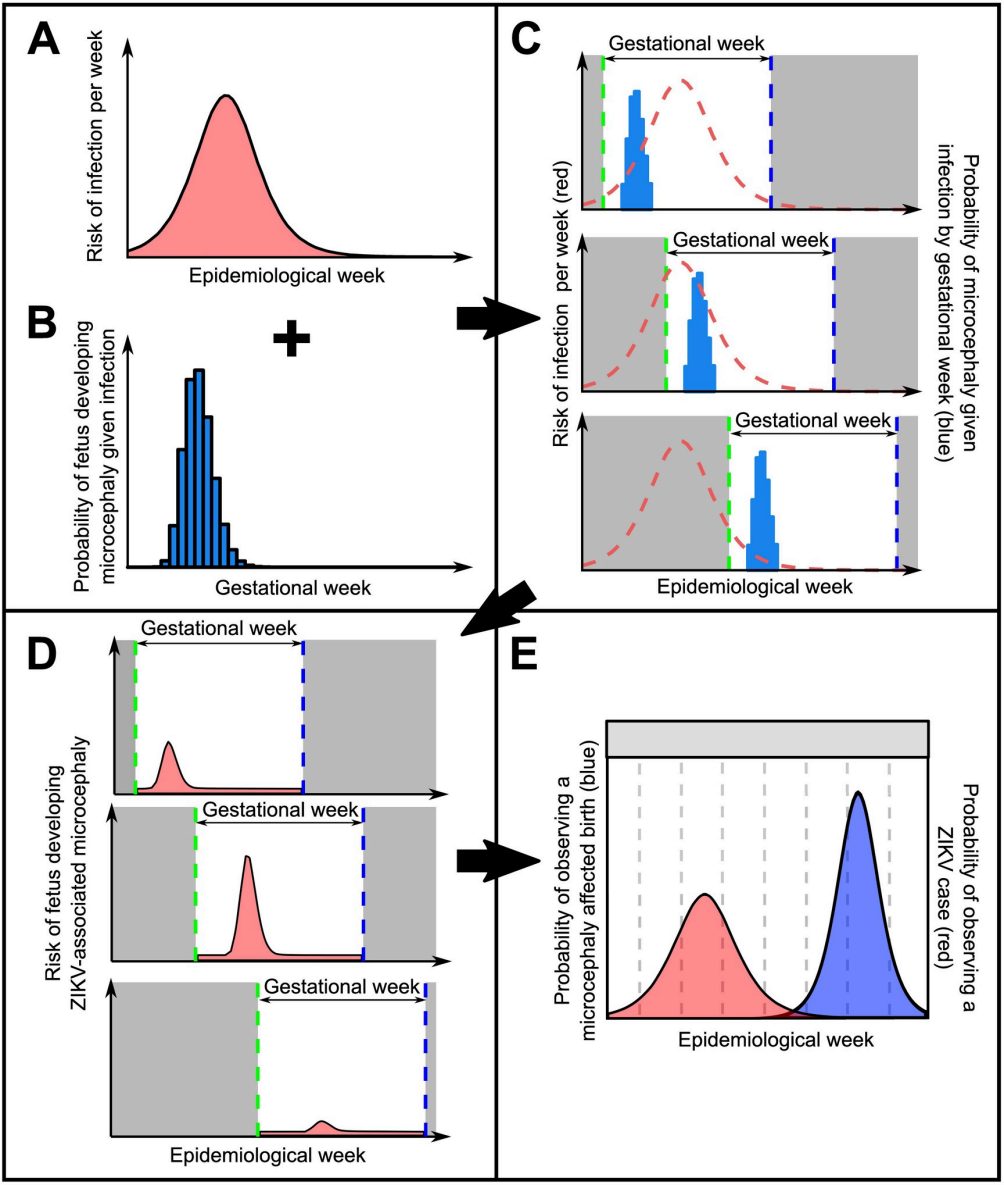
Schematic depicting the interaction of ZIKV epidemic dynamics and gestational-age-varying risk of microcephaly given infection. A: Time-varying risk of ZIKV infection generated from an SEIR model; B: probability of an individual fetus developing microcephaly given infection by week of gestation at time of infection; C: Link between risk of infection in real time and microcephaly risk in gestational time. Dashed red line shows time-varying risk of ZIKV infection, blue bars show microcephaly risk given infection, shaded grey region shows time before and after pregnancy, green dashed line shows conception time, blue dashed line shows expected birth date; D: Time-varying risk of microcephaly given ZIKV infection by epidemiological week for the same three example pregnancies, found by multiplying the risk of infection in a given epidemiological week (curve A) by the risk of microcephaly given infection in that week of gestation (curve B). Dashed blue line shows expected birth (observation) date; E: Combined probability of observing a microcephaly-affected birth in a given epidemiological week (blue), giving expected proportion of microcephaly-affected births. Red area shows probability of ZIKV infection by epidemiological week.

We developed a two-component model to describe the relationship between the incidence of ZIKV infection and the incidence of microcephaly-affected births, depicted in Fig 1. Full details of the model and sensitivity analyses are available in S1 Text and all code and analyses are available as an R package (https://github.com/jameshay218/zikaInfer). Our aim was to estimate the shape and size of the risk window for developing ZIKV-associated microcephaly given infection during gestation, and to test for differences in inferred risk using data sets from various Brazilian states and Colombia. The first component of the model described the transmission dynamics of ZIKV via the *Aedes aegypti* mosquito vector based on the Ross-MacDonald model for vector-borne disease. [27] Through estimation of the force of infection over time, we estimated a per capita risk of human infection per unit time, *P*_*I*_(*t*). The second component of the model described the risk of a fetus developing microcephaly given that the mother was infected in a particular week during pregnancy using a modified gamma function, $P_{m}^{\prime }\left(t\right)$. The expected proportion of microcephaly-affected births at any time *t* (Fig 1E) was obtained by multiplying these two components together:

$$\begin{array}{c} P_{m}\left(t\right)=\sum_{i=t-40}^{t}P_{I}\left(i\right)P_{m}^{\prime }\left(i-t+40\right) \end{array}$$ (1)

where *P*_*m*_(*t*) is the probability of a ZIKV-associated microcephaly birth at time *t*, *P*_*I*_(*i*) is the probability of an individual becoming infected at time *i* (and not before), and $P_{m}^{\prime }\left(i-t+40\right)$ is the probability of a fetus developing microcephaly given ZIKV infection at gestational week *i* − *t* + 40. Including a baseline microcephaly rate gives the probability of observing any microcephaly case at time *t* as:

$$\begin{array}{c} P_{micro}\left(t\right)=\phi_{i}\left[P_{m}\left(t\right)+P_{b}-P_{b}P_{m}\left(t\right)\right] \end{array}$$ (2)

where *P*_*b*_ is the baseline per birth microcephaly incidence rate and *ϕ*_*i*_ is a multiplicative factor for the number of true cases that were reported in location *i* (less than one indicates underreporting, greater than one indicates overreporting).

### Model parameters and fitting

Model parameters governing ZIKV transmission were obtained from the literature as described in S2 Table. Fixed parameters were mainly chosen based on a previously published transmission model resulting in a generation time (ie. the length of time between the time of infection in a human case and the times of infection in the secondary human cases resulting from that case) of approximately 20 days. [17] Given a fixed generation time, the model allowed the shape of the incidence curve for number of infected individuals (ie. the number of individuals entering the I compartment of the SEIR model) to vary depending on the value of the basic reproduction number, *R*_0_. As *R*_0_ is comprised of multiple correlated parameters, all components of *R*_0_ other than the vector density per human were fixed. Vital statistics (human life expectancy and population size) for particular locations are described in S3 Table.

We calculated the combined likelihood of observing ZIKV and microcephaly incidence data conditional on the model parameters, as described in S1 Text. Where full ZIKV infection incidence data were not available (ie. Pernambuco, which only reported 3 weeks of ZIKV infection incidence in the first half of 2015), we fit the two-component model to microcephaly incidence data alone and placed a 4-month-wide window on the time of peak ZIKV infection incidence given by the model as a uniform prior centered on the time of peak reported ZIKV incidence in Pernambuco (ie. the peak of the three reported weeks). We fit the model separately to available notified and confirmed incidence data for each location using a Markov chain Monte Carlo (MCMC) framework written in R and C++ with ordinary differential equations solved used the rlsoda package. [28, 29] By examining the posterior distributions of the parameters that determined the gamma risk profile, we also estimated the following parameters of interest: first week of gestational age where ZIKV infection confers a risk of microcephaly greater than 1 in 1000; last week of gestational age where ZIKV infection confers a risk of microcephaly greater than 1 in 1000; number of gestational weeks spent at risk; mean per-trimester risk; gestational week of greatest risk (gamma mode).

We carried out a sensitivity analysis using seroprevalence data from the city of Salvador in Bahia, Brazil to infer the microcephaly risk profile without the SEIR component of the model. Here, we assumed that reported acute exanthematous illness (AEI) was proportional to the true incidence of ZIKV infection during this time, and scaled the weekly reported incidence to give a final attack rate of between 59.4% and 66.8% in line with ZIKV IgG seroprevalence estimates for Salvador in May 2016 based on an NS1 antigen ELISA. [30, 31]

### Explaining the presence or absence of second waves of microcephaly incidence

**Fig 2:**
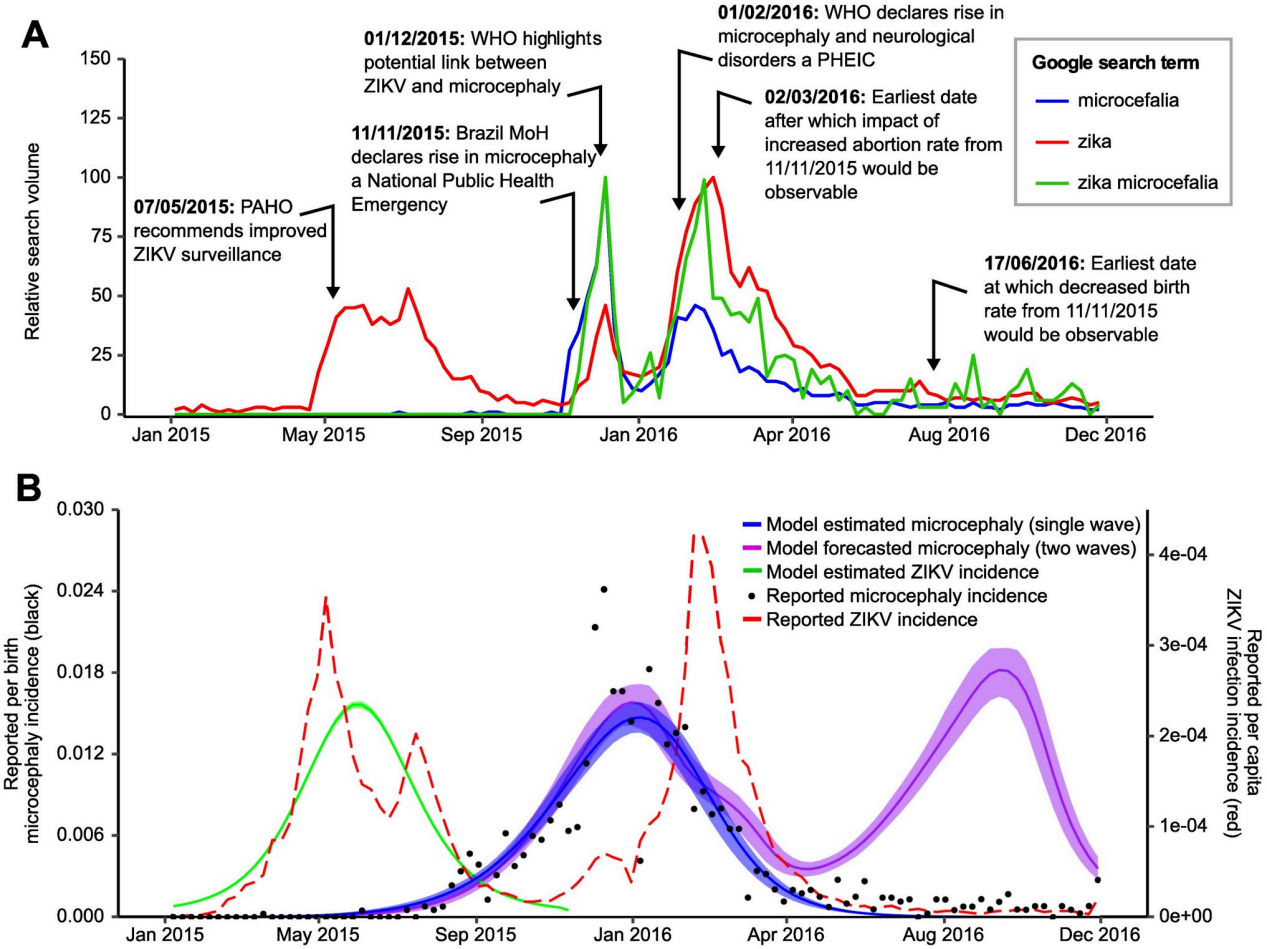
**A: Google search trends from 2015 to 2017 from Bahia, Brazil** [38] Y-axis shows the relative number of searches normalised by all searches at that time and location, with 100 indicating the highest search volume for that term across the entire time period. Red dashed lines highlight key epidemiological alerts that may have influenced public awareness and behaviour. MoH = Ministry of Health; PHEIC = Public Health Emergency of International Concern. **B: Model fits to the first wave of ZIKV and microcephaly incidence plotted with reported ZIKV and microcephaly incidence in Bahia, Brazil**. Red dashed line shows weekly reported ZIKV infection incidence per capita; green line shows per capita ZIKV infection incidence predicted by the SEIR model based on the maximum a posteriori probability (MAP) parameter estimates from fitting to the first wave of ZIKV infection incidence; black dots show reported weekly microcephaly incidence per live birth; blue line and shaded region show the model estimated MAP and 95% credible intervals (CI) on microcephaly incidence per live birth based on ZIKV infection incidence predicted by the SEIR model fitted to the first wave of microcephaly incidence; purple line and shaded region shows MAP and 95% CI forecasted per live birth microcephaly incidence assuming that the infection-risk relationship as estimated in Fig 4 persisted through the second wave, that reported ZIKV infection incidence represents the true incidence for both waves and that case reporting remained the same from 2015 to 2016.

We included four model parameters to quantify potential changes in behaviour and reporting rates that could explain the two seasons of observed data in Bahia, Brazil, where only one wave of microcephaly incidence was observed despite two waves of ZIKV infection incidence. Time-dependent changes in behaviour and reporting were likely during the epidemic due to media hype and public awareness, demonstrated by changes in Google search behaviour shown in Fig 2A. First, we assumed that microcephaly reporting became 100% accurate from March 2016 (the most recent change in case definition in Brazil) and estimated the relative reporting rate prior to this as a model parameter. [18, 32] Second, we assumed that immediately following the WHO declaration of a Public Health Emergency of International Concern in February 2016, the rate of aborted pregnancies under 24 weeks gestation could have changed. [33–35] Third, we assumed that the number of ZIKV-affected births after this date may have changed, either due to avoided pregnancies or additional precautions taken by pregnant women to avoid infection relative to the rest of the population. [34, 35] Finally, we assumed that ZIKV infection reporting accuracy may have changed after 11/11/2015, when the Brazilian Ministry of Health declared a National Public Health Emergency, and just before WHO/PAHO issued an alert with laboratory detection guidelines for ZIKV. [36, 37]

Our model did not explicitly include seasonality, and the SEIR model was therefore only suitable for the single-season analyses. We therefore did not use the SEIR model component in the multi-season analysis, but rather assumed that the per capita risk of becoming infected with ZIKV was proportional to reported ZIKV infection incidence and that reported incidence of ZIKV infection represented a fraction of true cases scaled by one parameter up to 11/11/2015 and another from 11/11/2015 onwards.

## Results/Discussion

### Patterns of incidence

**Fig 3:**
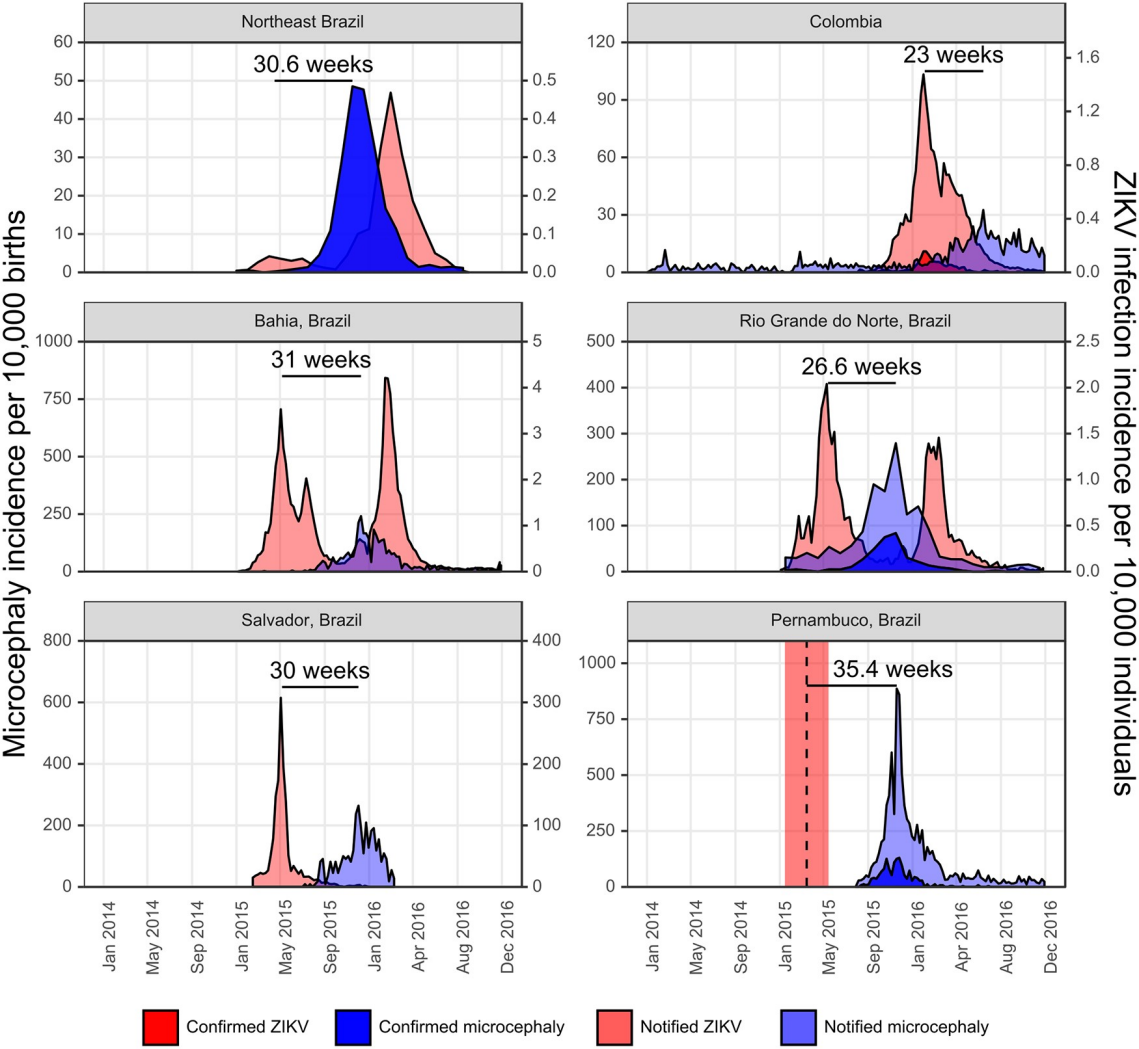
Notified and confirmed microcephaly and ZIKV infection incidence. X-axis shows date of report. Y-axis shows microcephaly incidence per 10,000 births (left) and ZIKV infection incidence per 10,000 individuals (right). Note different y-axis scales. ZIKV infection incidence for Northeast Brazil uses only reported cases in pregnant women with the entire population as the denominator. For Pernambuco, where comprehensive ZIKV infection incidence was not available, we show the peak epidemiological week from which incidence was reported (black dashed line) with a 4-month window (shaded red). Horizontal lines and labels show time in weeks between the initial peak of ZIKV infection incidence and peak microcephaly incidence. Confirmed and notified cases are distinguished by shading, where available. All incidence was reported by epidemiological week, apart from Northeast Brazil incidence and Rio Grande do Norte microcephaly incidence, which were reported by month. Note that no second y-axis is shown for Pernambuco, as no ZIKV infection incidence is shown.

Although our study data were from similar reporting systems, Fig 3 illustrates substantial differences in the key features of both ZIKV infection and microcephaly incidence patterns. Peak timing, width of the incidence curves, maximum per capita incidence and the lag between ZIKV infection and microcephaly incidence peaks differed by location and dataset. Variation in total and maximum per-birth microcephaly incidence indicates location-specific differences in the proportion of pregnant women that were infected with ZIKV or in the probability of developing microcephaly following infection. For example, weekly notified microcephaly incidence peaked at 32.6 cases per 10,000 births in Colombia, which was far lower than peak notified microcephaly incidence in Pernambuco, Brazil at 760 cases per 10,000 births, suggesting that microcephaly risk given infection and/or ZIKV attack rates were higher in Pernambuco. The lag between incidence peaks also varied, ranging from 23 weeks (bootstrapped confidence intervals: 19-32 weeks) for Colombia compared to 31 weeks (bootstrapped confidence intervals: 30-36) from state-level reports for Bahia, Brazil. Given that only a small fraction of the Colombian population is at risk of arbovirus infection compared to the Brazilian population due to differences in vector ecology, it is unsurprising that the absolute per capita incidence of ZIKV infection and microcephaly are lower in Colombia than in Brazil. [22] However, as the population at risk of ZIKV infection is the same population at risk of microcephaly, we would expect the lag and relative magnitudes of ZIKV infection and microcephaly incidence to be the same between Brazil and Colombia. These differences in time lags and relative magnitudes therefore suggest that the time of peak risk during pregnancy may have varied between locations, potentially through differences in additional risk factors in these locations such as prior arbovirus exposure.

Differences in observed incidence patterns may also arise as a result of reporting bias, which may be reduced using confirmed rather than notified case data. Although some confirmed microcephaly case data were available for Brazil, the only available data of confirmed microcephaly cases in Colombia reported number of cumulative confirmed cases. [39] It should also be noted that only a subset of suspected cases were laboratory confirmed in Colombia to test for the presence of ZIKV in municipalities which had yet to confirm ZIKV infection, and case reporting was otherwise based on clinical symptoms. [24] Due to variation in confirmation delays, we were unable to extract the time of birth of these cases and were therefore unable to use these data in model fitting. Between 03/01/2016 (epidemiological week (EW) 1 of 2016) and 05/06/2017 (EW 18 of 2017), approximately 70% of notified microcephaly cases were discarded in Colombia (328 cases confirmed, 874 discarded, 37 under investigation), highlighting that total notified case data likely overestimates true incidence. [40] The proportion of total notified cases that were discarded was similar for Rio Grande do Norte (138 confirmed vs. 475 notified) and somewhat higher for Pernambuco (365 confirmed vs. 2117 notified). Confirmed ZIKV infection incidence were available for Colombia but not Brazil due to the lack of reporting infrastructure during the first wave. [41] The lag between peak ZIKV infection and microcephaly incidence did not change when using notified or confirmed ZIKV infection or microcephaly data.

### Different data sets suggest different risk profiles

**Fig 4:**
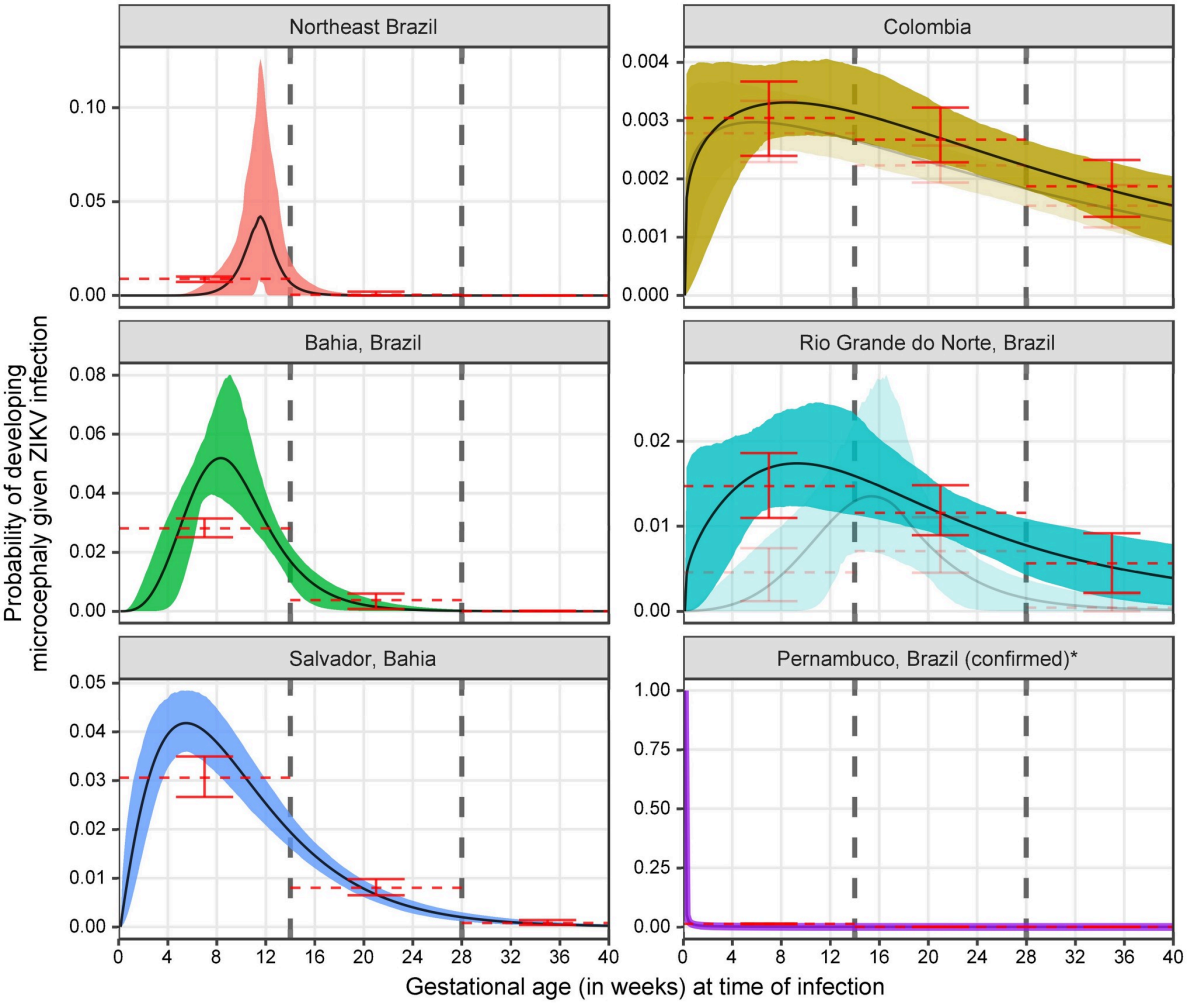
Estimates of the microcephaly risk profile fit to different data sets. Parameter estimates corresponding to these risk profiles are provided in S4 Table. Note different y-axis scales. Shaded regions show 95% credible intervals, black lines show posterior means. Vertical dashed lines show boundaries of the first, second and third trimester. Horizontal red dashed lines show the mean estimate for the mean model-derived risk (ie. average of the model suggested risk for that trimester) during each trimester and red bars show 95% credible intervals. Data sets were as follows (left-right). A: Notified ZIKV infections in pregnant women and confirmed microcephaly incidence data from the entirety of Northeast Brazil combined. [42] B: Notified ZIKV infection and microcephaly incidence data from Colombia (confirmed ZIKV faded). [43] C: Notified ZIKV infection and microcephaly incidence from Bahia, Brazil state-level reports. [44] D: Notified ZIKV infection and microcephaly incidence from Rio Grande do Norte, Brazil state-level reports (confirmed microcephaly faded). [45, 46] E: Notified AEI and microcephaly incidence from Salvador, Bahia, Brazil. [30] F: Confirmed microcephaly incidence from Pernambuco, Brazil state-level reports. [47]. Note that the model is unable to explain the observed data with a biologically interpretable risk profile, suggesting substantial bias in the data.

We inferred interpretable gestational risk profiles for 5 of the 6 datasets used here (Fig 4). Though clear estimates of the gestational-age-varying risk were obtained for each location, substantial differences are apparent in the inferred gestational age of peak risk, duration of the risk period, and maximum absolute risk. The model did not produce a biologically interpretable risk profile using data from Pernambuco, Brazil that was comparable to those inferred using the other 5 datasets. Sensitivity analyses excluding the ZIKV infection incidence data from the model fitting for other locations were able to produce plausible risk profiles, suggesting biases in reported microcephaly incidence data for Pernambuco that could not be explained by the model (see Section 4.3, S1 Text). It is important to note that microcephaly is only one manifestation of CZS, and the risk profile of other adverse outcomes may differ. These risk estimates therefore apply only to the specific outcomes of proportionate and disproportionate microcephaly, which were not distinguished in these data. [8]

### Gestational age at peak risk

The time from the peak of ZIKV infection incidence to the peak of microcephaly incidence indicates the typical gestational age at which microcephaly cases were infected. When using both state and city level notified ZIKV infection and microcephaly incidence from Bahia, Rio Grande do Norte, and Salvador, Brazil, the peak week of gestational-age-varying risk was estimated to be in the middle of the first trimester (Fig 4). When notified ZIKV infection incidence in pregnant women and confirmed microcephaly incidence from Northeast Brazil were used, the estimated peak gestational-age-varying risk was towards the end of the first trimester. Notified case data from Colombia were also suggestive of peak risk in the first trimester. Inference did not change substantially using confirmed as opposed to notified ZIKV infection incidence data for Colombia; however, using confirmed microcephaly incidence data for Rio Grande do Norte resulted in a shift of the risk profile towards the start of the second trimester. When a ZIKV infection incidence peak in a 4-month window around March 2015 was assumed for Pernambuco, the inferred microcephaly risk profile was highly skewed towards the first week of pregnancy, suggesting that these data are incompatible with the other 5 data sets.

### Duration of heightened gestational risk

There was substantial variation in the inferred window of heightened gestational risk between different populations. The window of heightened gestational risk is estimated from the relative durations of the ZIKV infection and microcephaly incidence curves (using an illustrative threshold of 1 in 1,000 infections leading to microcephaly to define the heightened gestational risk window). A narrow period of ZIKV infection incidence preceding a wide period of microcephaly incidence suggests a wide window of heightened gestational risk. If the period of heightened gestational risk is long, then infections at a particular point in time would present as cases of CZS across a wider interval of birth dates. Inferred risk profiles using notified case data from Rio Grande do Norte, the city of Salvador and Colombia all suggested heightened risk throughout pregnancy (Fig 4).

Conversely, two similarly narrow (or wide) periods of ZIKV infection and microcephaly incidence would suggest a relatively small window of heightened gestational risk, as all ZIKV-affected pregnancies would present as births after a similar delay. A true microcephaly incidence period that is narrower than the ZIKV infection incidence period should not be possible, as the narrowest microcephaly incidence curve would arise when all infected pregnant women give birth after the same delay. Aggregated confirmed case data from Northeast Brazil, state-level notified case data from Bahia and state-level confirmed case data from Rio Grande do Norte all suggested a more limited window of risk during pregnancy, with lower risk suggested towards the end of pregnancy (Fig 4, Northeast Brazil, Bahia and Rio Grande do Norte (confirmed cases)).

Public awareness, media hype, changing criteria for case reporting and variation in laboratory testing capacity likely resulted in changing reporting rates throughout the epidemic. [18, 41, 48, 49] Location-specific time-varying changes in reporting sensitivity and specificity are therefore one potential explanation for differences in the risk profiles inferred using data from Northeast Brazil and Colombia. Given that Colombia was expecting an increase in microcephaly cases during 2016, an increase in notified cases may have been reported before a true increase in confirmed cases, which would falsely suggest some gestational risk late in pregnancy. Time-varying reporting bias may also explain the extremely narrow and early window of risk inferred using data from Pernambuco (Fig 4, Pernambuco, Brazil). The impact of reporting bias is clearly demonstrated by the contrasting results using confirmed or notified microcephaly case data for Rio Grande do Norte, wherein confirmed data suggested a narrower and later risk window than the notified data.

### Absolute risk of CZS

The absolute risk of CZS is more difficult to estimate as it depends on the true incidence of ZIKV infection in pregnant women and CZS cases as a proportion of live births. A high ZIKV infection attack rate with known microcephaly incidence would suggest a lower microcephaly risk per infection to the fetus than a low infection attack rate with the same observed microcephaly incidence. [7] Reported infection incidence data may be subject to under-reporting and over-reporting, potentially through missing asymptomatic or mild cases that might not present to surveillance systems (under-reporting), or misclassifying infections caused by other arboviruses as ZIKV infection, namely dengue and chikungunya virus (CHIKV) (over-reporting). [41, 50] These confounders present identifiability problems in inferring levels of true incidence and therefore microcephaly risk; surveillance data in a scenario of high risk with under-reporting would be similar to a scenario of low risk with over-reporting. For example, during the 2015 wave in Brazil many cases of illness likely caused by ZIKV were misclassified as dengue infection, resulting in under-reporting of ZIKV infection incidence. [41] Over-reporting of microcephaly incidence during the initial wave of cases was also possible, due to changing case definitions, reclassification of suspected cases and increased awareness in surveillance systems. [18, 32] Estimating the proportion of true ZIKV infections that led to observed microcephaly cases is therefore dependent on knowing the true risk of ZIKV infection during the epidemic period. ZIKV IgG seroprevalence was estimated to have reached 63.3% (95% confidence interval, 59.4 to 66.8%) in Salvador, Brazil between 2015 and 2016 despite only 16,986 reported cases of AEI from a population of nearly 3 million (approximately 0.6%), suggesting that under-reporting of ZIKV infection incidence was a key problem in this location. [30, 31]

By assuming that 100% of true microcephaly cases were reported but that reported ZIKV cases represented only a fraction of the true incidence, we inferred the absolute risk of ZIKV-associated microcephaly from each of the datasets (S4 Table). The average first trimester risk of microcephaly given ZIKV infection was estimated to be 2.81% (mean; 95% credible interval (CI): 2.51-3.16%) based on data from Bahia, Brazil, but much lower in the second trimester at 0.365% (mean; 95% CI: 0.0715-0.588%). Conversely, the level of absolute risk estimated using notified case data from Colombia suggested that the risk was lower but consistent throughout gestation at 0.303% (mean; 95% CI: 0.239-0.367%), 0.268% (mean; 95% CI: 0.228-0.322%) and 0.186% (mean; 95% CI: 0.135-0.232%) in the first, second and third trimesters respectively. The former estimate is slightly higher than risk estimates inferred based on seroprevalence data from French Polynesia which suggested a risk of 0.95% (95% confidence interval; 0.34–1.91%) in the first trimester, whereas the latter estimate suggests a lower risk. [6]

We performed a sensitivity analysis with better constraint on the true ZIKV attack rate by taking microcephaly and AEI data from Salvador, Brazil for 2015 scaled by recent ZIKV IgG seroprevalence data, as described in Section 6, S1 Text. [30] Here, we assumed that the true risk of ZIKV infection in Salvador was proportional to the per capita reported incidence of AEI scaled such that the overall attack rate was between 59.4% and 66.8%. [31] Based on the ZIKV infection and microcephaly incidence data from Salvador, Brazil, we estimated the mean first trimester risk of microcephaly given ZIKV infection to be 3.06% (mean, 95% CI: 2.66-3.49%); the mean second trimester risk to be 0.805% (mean, 95% CI: 0.649-0.980%); and the mean third trimester risk to be 0.0833% (mean, 95% CI: 0.0407-0.142%). We did not scale incidence data for any other location due to the lack of seroprevalence data. However, given that the model is powered by the pattern of microcephaly incidence relative to the pattern of ZIKV infection incidence after accounting for differences in infection risk and reporting, these risk estimates may apply to other locations if no additional cofactors affect the risk of microcephaly given infection.

### Understanding the missing second wave of microcephaly incidence

Despite a clear second wave of GBS incidence at the beginning of 2016, no second wave of microcephaly incidence in Northeast Brazil was observed in the latter half of 2016. [11] Similar to [11], Fig 2B illustrates the incidence of microcephaly that would have been expected in Bahia, Brazil using our model framework and based on reported ZIKV infection incidence under the assumption that the underlying gestational-age-varying risk profile and reporting behaviour did not change from 2015 to 2016.

We used the population-level data fitting framework described above to test the hypothesis that plausible changes in behaviour or reporting are sufficient to provide a consistent narrative between the two waves of ZIKV and microcephaly case data. Fig 2A describes the timings of particular events that may have led to these changes. We considered four hypotheses describing changes in behaviour and reporting rates. First, we assumed that microcephaly reporting accuracy may have been different before week 11 of 2016 (13/03/2016, the most recent change in case definition in for microcephaly reported through the Registro de Eventos em Saúde Pública (RESP) database in Brazil) [32, 51] and estimated the relative reporting rate for microcephaly prior to this that would be consistent with the observed data. Second, we assumed that immediately following the National Public Health Emergency announcement by the Brazilian Ministry of Health on 11/11/2015, the frequency of early abortions (up to 24 weeks gestation) due to early detection of CZS may have increased. [36] The earliest date at which targeted abortions would be observed as a drop in birth rate would be 16 weeks after this shift in behaviour (02/03/2016). [34, 35, 52] A reduction in birth rate from delayed pregnancy would also be possible; however, this would only appear approximately 40 weeks after the behavioural shift. Third, we assumed that the number of pregnant women affected by ZIKV after this date may have changed through additional precautions taken to avoid infection relative to the rest of the population. [53] Finally, we assumed that ZIKV reporting itself may have changed on 11/11/2015 before the start of the second wave of ZIKV infection incidence through increased surveillance, increased awareness and/or increased misclassification of other arbovirus infections as ZIKV infection. Over both time periods, we assumed that the per capita risk of becoming infected with ZIKV was proportional to reported ZIKV infection incidence, but that the scale of that proportion changed on 11/11/2015 following the potential change in ZIKV infection reporting.

**Fig 5:**
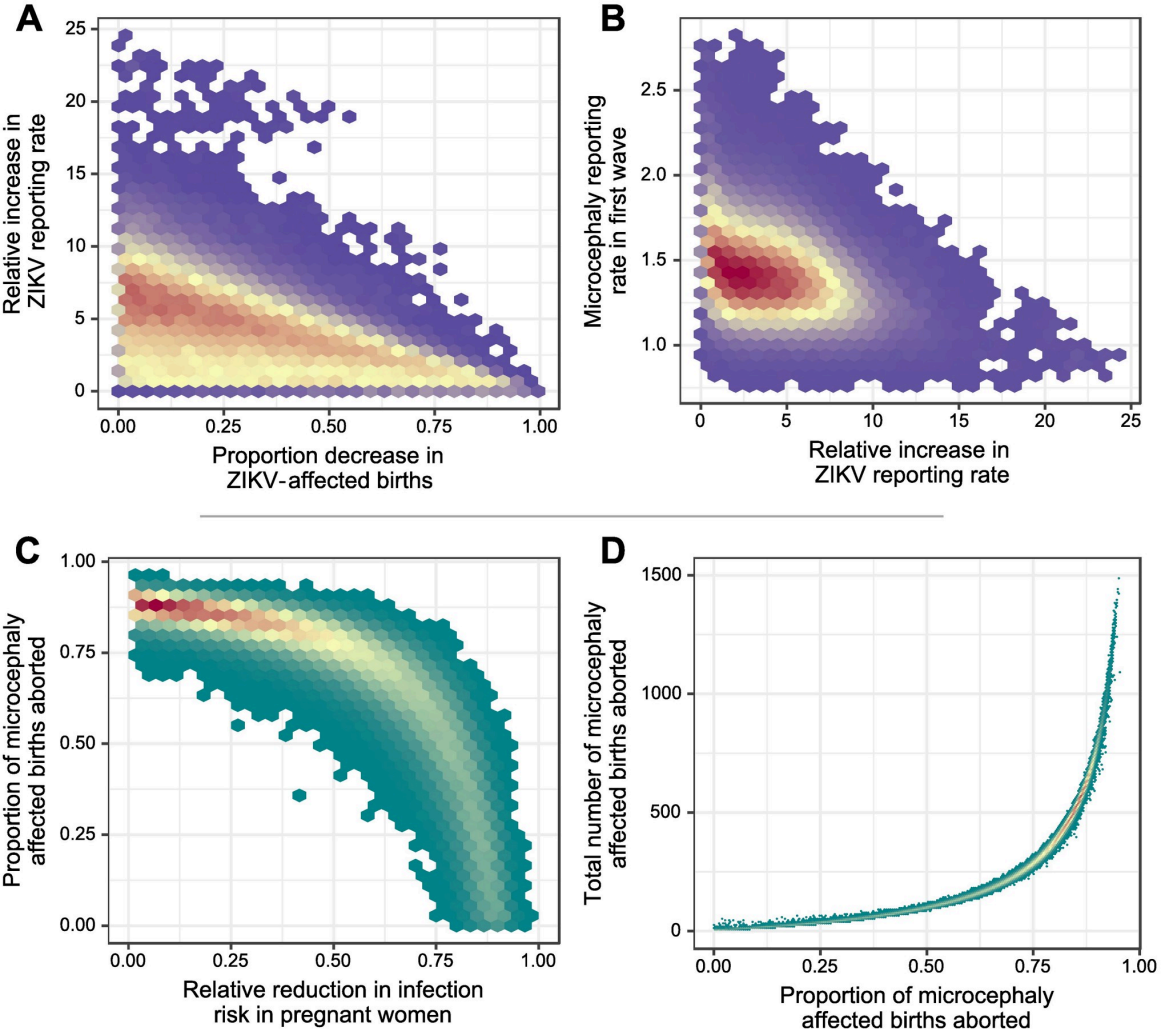
Key regions of parameter space that are consistent with the observed data. Unshaded regions show areas of parameter space that are less consistent with the observed data. Two-dimensional posterior density estimates for the behavioural and reporting changes necessary to explain the lack of a second microcephaly incidence wave in Bahia, Brazil. Parameter estimates are provided in S5 Table. Better supported regions of parameter space are indicated in red/orange, whereas less well supported regions are purple/turquoise. Plots A and B were estimated assuming that ZIKV reporting behaviour could have changed between the two waves, whereas plots C and D were estimated assuming that ZIKV reporting behaviour stayed the same throughout the epidemic. All estimates presented assumed that microcephaly reporting, targeted abortions and infection avoidance behaviour may have been present in the second wave, as described in the main text. A: Negative correlation between the increase in ZIKV reporting and increase in abortion rate, suggesting that the observed data could be explained by either mechanism in the absence of the other. B: Lack of correlation between ZIKV reporting and microcephaly reporting change estimates suggest that both mechanisms may independently explain observations. C: High correlation between the proportion reduction in ZIKV-affected pregnant women and the proportion of targeted abortions assuming no change in ZIKV reporting between the two waves, suggesting that high levels of either, or moderate levels of both mechanism are required to explain the data. D: Relationship between total aborted births between 02/03/2016 and 31/12/2016 and the proportion of ZIKV-associated microcephaly-affected births aborted, highlighting the actual number of aborted pregnancies that would have occurred given a particular abortion rate.

Based on state-level reports from Bahia, Brazil and assuming that ZIKV infection reporting did not change, our analyses suggest that the lack of a second microcephaly peak could be explained by the combined effect of: a 151% reporting rate of microcephaly cases prior to 13/03/2016 relative to fixed 100% accurate reporting after 13/03/2016; targeted abortions ending 88.4% of microcephaly-affected pregnancies prior to 24 weeks gestation; and a relative decrease in infection probability in pregnant women of 0.60% (values shown are the maximum a posteriori probability (MAP) estimates). It is important to note that many of these parameters are highly correlated, suggesting that these data could be explained by a combination of multiple mechanisms, or by a greater contribution of some mechanisms and a reduced effect from the others (Fig 5). If ZIKV infection reporting accuracy increased substantially between the two waves in addition to the behavioural changes described above, then a smaller increase in the proportion of terminated pregnancies would have been necessary. Similarly, targeted abortions and precautions to avoid infection by pregnant women would present a similar reduction in microcephaly incidence, and these estimates are therefore highly correlated (Fig 5C).

Assuming that there were no targeted abortions, no additional precautions to avoid infection taken by pregnant women, and no change in microcephaly reporting accuracy, we estimated that these data could be explained solely by a 18.9-fold (mean, 95% CI: 10.0-59.1-fold) increase in ZIKV infection reporting after 11/11/2015. Conversely, assuming that targeted abortions after 11/11/2015 were the only change, 92.5% (mean, 95% CI: 89.8-94.9%) of microcephaly-affected births would need to have been aborted to explain the lack of a second peak, corresponding to 1090 (803-1480) aborted pregnancies between 02/03/2016 and 31/12/2016. Fig 5D shows how the total number of aborted microcephaly-affected births, which may be observable, would change with different abortion rates of microcephaly-affected births. If microcephaly reporting accuracy were the only factor to change, then a 601% (mean, 95% CI: 492-726%) reporting rate of microcephaly cases prior to 13/03/2016 relative to fixed 100% accurate reporting after 13/03/2016 would have been necessary. Accurate data on the true number of abortions in this time period and information on the changes in ZIKV and microcephaly reporting would help to clarify the relative contributions of these mechanisms.

### Remaining uncertainty and future work

Overall, these results highlight the limitations of currently publicly available population-level data in explaining epidemiological trends. Different datasets suggest different risk profiles, some of which contrast with previous population-scale analyses. Whilst data from Bahia, Brazil were suggestive of a risk profile similar to that estimated using data from French Polynesia, data from Colombia and Rio Grande do Norte, Brazil suggest a much longer gestational risk period. [6]

Although reporting bias may explain the differences in inferred microcephaly risk in different locations, heterogeneity in the distribution of additional host risk factors of microcephaly may be important. Interpretation of epidemiological data for dengue infection requires an understanding of pre-existing immunity due to the presence of antibody-dependent enhancement, which may also be relevant to the interpretation of CZS incidence given the potential role of dengue antibodies in ZIKV disease enhancement. [54–56] Observations of increased prior dengue exposure in areas of disproportionately increased microcephaly incidence would support this hypothesis and be of importance for dengue- but not yet ZIKV-affected areas, highlighting the need for comprehensive serological studies. [31, 57] An understanding of other potential host risk factors that may differ between affected areas, such as socioeconomic status or maternal smoking, will further aid the interpretation contrasting incidence data. [32]

A limitation of our model is the aggregation of data into high-level administrative units, which may mask small-scale heterogeneity in infection risk and case reporting. This may be particularly problematic in our analysis for Colombia, as using the entire Colombian population and birth numbers as the susceptible population may underestimate the true risk should only a fraction of the population actually be exposed to ZIKV infection. [58, 59] Similarly, differences in transmission peak times at a small spatial scale coupled with location-specific reporting accuracy may reduce the reliability of the population-wide inferred risk profile. Although we were unable to fit the model at a smaller administrative unit due to the lack of necessary meta-data for Colombia, doing so may reveal a similar risk profile to that estimated using data from Northeast Brazil.

Our estimates suggest that ZIKV infection reporting rates would need to have increased 18.9-fold (mean, 95% CI: 10.0-59.1-fold) to explain the lack of a second microcephaly wave in Bahia, Brazil on its own, which may have been possible if awareness and diagnostic accuracy improved through the epidemic. We note that syndromic ZIKV reports may have included misclassified CHIKV infections which may not have represented an increased risk of ZIKV-associated microcephaly during the second wave in 2016. [60] A 18.9-fold increase in ZIKV reporting as estimated here could therefore mean that ZIKV reporting was a more accurate representation of the true ZIKV attack rate in 2016, or that 18 Chikungunya cases were misclassified as ZIKV for every 1 true reported ZIKV case with no change in the proportion of true ZIKV cases that were reported. [41] However, during the period in which second waves of ZIKV infection occurred, there was sufficient virological testing to justify confidence in the relative specificity of reported ZIKV cases. [61] Furthermore, in Salvador, Brazil, where serological data are available, the increase in CHIKV seropositivity from 2015 to 2016 was far lower than for ZIKV seropositivity. [31] Nonetheless, diagnostic tools with improved sensitivity and specificity in distinguishing these infections would help to clarify the proportion of true ZIKV infection incidence that observed incidence data represent.

We estimated that 1090 (mean, 95% CI: 803-1480) microcephaly-affected births would need to have been aborted between 02/03/2016 and 31/12/2016 to explain the observed data through increased abortions alone. Given that approximately 1000 abortions are reported in Northeast Brazil weekly, it may be possible to identify the true increase in abortion rate during this time period if and when complete data become available (Supplementary Material of [11]). [35]

Estimating the true shape and magnitude of the underlying gestational-age-varying risk profile requires additional data that could either be gathered retrospectively or through surveillance in areas where the first wave of transmission is ongoing or has not yet happened. A key limitation of the epidemiological data gathered in Brazil during 2015 and early 2016 is that surveillance systems were implemented during the epidemic, leading to possible inconsistencies in case definitions and ascertainment rates. Retrospective regional serological surveys have been suggested previously as a means of inferring attack rates, which would constrain estimates for the reporting rate of microcephaly and ZIKV infection and in turn constrain estimates of both the underlying risk and potential changes in behaviour/reporting in the second wave. [60, 62] In particular, community seroprevalence studies of ZIKV antibodies in women of child-bearing age would provide an accurate estimate of the true proportion of ZIKV-infected women during the outbreak irrespective of symptomatic status and time of infection. In terms of future outbreaks, consistent and accurate case definitions for microcephaly and CZS,—such that sensitivity and specificity are high throughout the epidemic period—would greatly increase the utility of clinical surveillance data for population-level analysis.

A key remaining question is whether or not the epidemiological data from Brazil accurately represent the relationship between ZIKV infection and microcephaly, and indeed the wider set of outcomes associated with CZS. Retrospective cohort studies for women of childbearing age to assess whether changes in behaviour regarding conception and infection avoidance occurred in 2016 should clarify whether the second season of ZIKV/microcephaly in Brazil is fully consistent with estimates of gestational-age-varying risk from the first season. [53] If actual reporting rates and behaviour changes are not sufficient to explain the apparent discrepancy between first-wave incidence in Brazil compared to later and elsewhere, the investigation of other potential cofactors, such as prior arbovirus infection, becomes a higher priority. It should then be possible to accurately calculate the risk of CZS based on gestational age at infection and the presence or absence of other possible cofactors.

## Supporting information

S1 Text Full detailed description of the model, data and sensitivity analyses. (PDF)

S1 Table Summary of datasets included in the analysis. For each location, the type of incidence data, whether reported data were confirmed or all notified cases, the case definitions used, the relative file location in the accompanying R package, the time resolution of reports, whether or not data were extracted using a digitiser, and the data source are provided. Sources correspond to references in the main text.
(CSV)

S2 Table Summary of model parameters, sources and assumed parameter ranges. Parameter symbols are as described in S1 Text. The component column refers to which part of the model or which part of the analysis that parameter relates. Values shown are the fixed values used in the analysis if the parameter was not estimated. Where specified, lower and upper bounds refer to uniform prior ranges imposed during the MCMC fitting. Sources correspond to references in the main text.
(CSV)

S3 Table Summary of vital statistics and sources. Sources correspond to references in the main text.
(CSV)

S4 Table Summary of parameter estimates for the primary analyses based on estimated posterior distributions. Columns describe the name of the analysis as shown in Fig 4; the name of the parameter; and the posterior means and 95% credible intervals for those parameters.
(CSV)

S5 Table Summary of parameter estimates for the analysis of the second ZIKV infection incidence wave. Analysis column describes which mechanism was under investigation for that analysis; parameter column describes the parameter being estimated; values shown are the posterior mean and 95% credible intervals.
(CSV)
