# Supplementary material for: Potential inconsistencies in Zika surveillance data and our understanding of risk during pregnancy
Source: PLoS Negl Trop Dis. 2018 Dec 10;12(12):e0006991. doi: 10.1371/journal.pntd.0006991 (PMC6301717; doi:10.1371/journal.pntd.0006991)
Supplement: S1 Text — (PDF) [file pntd.0006991.s001.pdf]

Potential inconsistencies in Zika surveillance data and our understanding of risk during pregnancy

Supplementary Text S1

Contents

1 Introduction **1**

2 Model description **1**

2.1 Transmission model 2

2.2 Microcephaly risk model 5

2.3 Combined model 5

3 Data **6**

3.1 Microcephaly and ZIKV incidence data 6

3.2 Model parameters 9

4 Model fitting **10**

4.1 Microcephaly incidence likelihood 10

4.2 ZIKV incidence and combined likelihood 10

4.3 Model fitting without ZIKV incidence data 11

4.4 MCMC algorithm 12

5 Forecasting the second wave of microcephaly incidence **12**

5.1 Parameter estimation 14

6 Fitting to data from Salvador, Brazil **14**

References **16**

1 Introduction

This vignette describes the methodology and data behind the `zikaInfer` R package.

2 Model description

We developed a two-component model to describing the relationship between incidence of ZIKV infection and of microcephaly incidence, as depicted in Figure 1 in the main text.

2.1 Transmission model

We developed an SEIR model to capture the transmission dynamics of ZIKV via the *Aedes aegypti* mosquito vector, based on the Ross-MacDonald model for vector-borne disease, capturing deterministic SEIR dynamics in humans with transmission via the mosquito vector experiencing SEI dynamics.[1] Using a transmission model to approximate per capita infection risk rather than assuming that reported rates were equal to infection risk has two benefits:

1. Model fitting can be done based on information inherent in the shape of the curve (width, growth rate) rather than its magnitude. This means that, assuming the reported incidence is a good estimate of the shape of the epidemic curve even if the magnitude is not an accurate reflection of the true incidence (misreporting), we can fit the model to reported data. By adding an additional parameter to scale the magnitude of the model-predicted incidence curve (ie. proportion of true cases reported), we produce an estimate for the true time-varying risk of ZIKV infection.

2. By fixing some of the model parameters based on the literature (Table S2), we can infer values for the basic reproductive number, R_0, and the epidemic seed time, t_0.

The model is defined by the following set of ODEs:

$$\begin{aligned}
\tfrac{dS_M}{dt} &= \mu_M N_M - \mu_M S_M - \lambda_M S_M \\
\tfrac{dE_M}{dt} &= \lambda_M S_M - \sigma_M E_M - \mu_M E_M \\
\tfrac{dI_M}{dt} &= \sigma_M E_M - \mu_M I_M \\
\\
\tfrac{dS_H}{dt} &= \mu_H N_H - \lambda_H S_H - \mu_H S_H \\
\tfrac{dE_H}{dt} &= \lambda_H S_H - \sigma_H E_H - \mu_H E_H \\
\tfrac{dI_H}{dt} &= \sigma_H E_H - \gamma_H I_H - \mu_H I_H \\
\tfrac{dR_H}{dt} &= \gamma_H I_H - \mu_H R_H
\end{aligned} \tag{1}$$

Where S, E, I and R indicate the number of individuals in the susceptible, exposed, infected or recovered compartment, and the subscript represents either human (H) or mosquito (M) populations; N is the total population size; μ is the birth/death rate; σ is the latent period; γ is the infectious period; and λ is the force of infection. We assumed that each location (typically a Brazilian state) was a closed, homogeneously mixing population with constant population size.

Through calculation of the force of infection over time, we estimated a per capita risk of infection per unit time. The force of infection for mosquitoes and humans respectively is given by:

$$\begin{aligned}
\lambda_M &= b p_{HM} I_H \\
\lambda_H &= b p_{MH} I_M
\end{aligned} \tag{2}$$

Where b is the bite rate per vector; p_{MH} is the probability of a bite generating an infection in a human from an infected vector; p_{HM} is the probability of a bite generating an infection in a vector from an infected human; and I_H/I_M is the number of infected humans/mosquitoes.

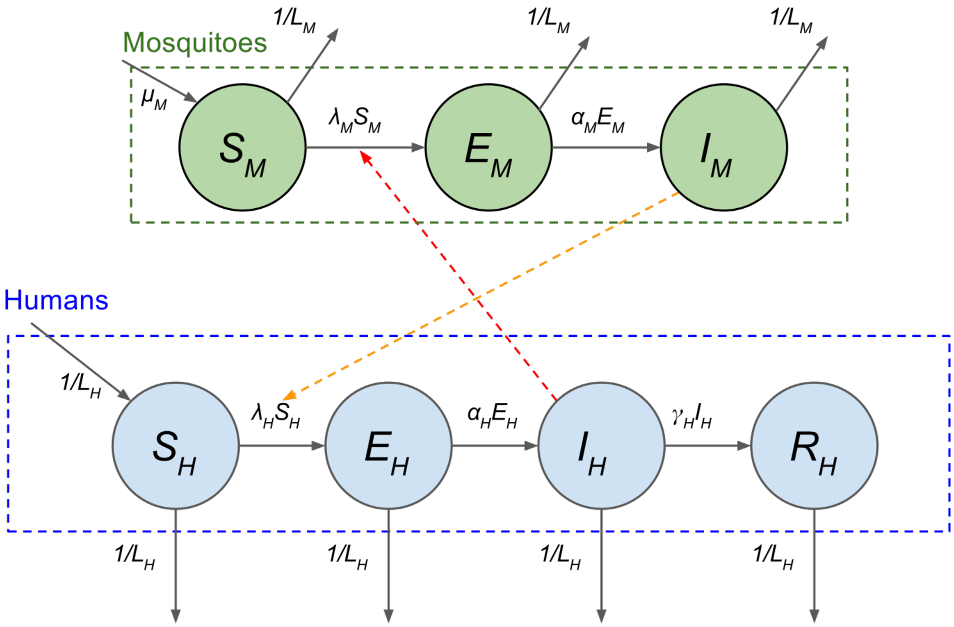

Figure 1: **Graphical representation of the SEIR model.** Mosquito vector population is shown in green, with new mosquitoes entering the susceptible class (S_M) and progressing through to the infected state. The human population is shown in blue, with new humans entering as susceptible (S_H). Humans become infected at a rate of λ_H, and become infectious at a rate of α_H. Humans then recover at a rate of γ_H. Note that the force of infection on humans comes from mosquitoes only, as represented by the orange arrows. All compartments experience a death rate of 1/L, where L is the lifespan in days.

Using this force of infection term, we defined the probability of an individual not becoming infected at a given time, t, as:[2]

$$f(t) = \exp(-\lambda_H(t)\delta t) \tag{3}$$

Where $f(t)$ is the probability of remaining susceptible between t and $t+\delta t$. Here, we used $\delta t = 1$ day (approximately $1/20$th of the assumed generation time) to approximate the probability of remaining susceptible within a small, discrete period of time. We validated this choice of time step by testing values for δt between 0.1 and 2, which did not affect the model results. Smaller values of δt were not used for computational reasons. We calculated the probability of remaining susceptible from t_0 up to a given time, t, as:

$$F(t) = \prod_{i=t_0}^{t} f(i) \tag{4}$$

Finally, the probability of becoming infected at a given period of time, t, was defined as:

$$P_I(t) = F(t)(1 - f(t)) \tag{5}$$

Where $P_I(t)$ is the probability of becoming infected between t and $t + \delta t$, given by the probability of remaining susceptible up to that point multiplied by the probability of not remaining susceptible during the small time period defined by δt. Note that $F(t)$ refers to the period of time up to t, whereas $f(t)$ refers to the period of time between t and $t + \delta t$. Note also that t is treated as a discrete unit here to approximate the theoretical relationship between time-varying force of infection and infection risk.[2]

The basic reproductive number, R_0, was defined as the number of new human infections generated by the introduction of a single infected human into a naive human and mosquito population given by:[3]

$$R_0 = \frac{b^2 p_{HM} p_{MH} N_M}{\mu_M(\sigma_M + \mu_M)(\gamma_H + \mu_H)N_H} \tag{6}$$

Where N_M is the total number of mosquitoes; N_H is the total number of humans; μ_M is the birth/death rate of mosquitoes; σ_M is the rate at which mosquitoes leave the exposed class; σ_H is the rate at which humans leave the exposed class; γ_H is the rate at which humans leave the infected class; and μ_H is the birth/death rate of the human population. Critical values for R_0 were used to validate model implementation (values at, just above and below 1). We also validated the use of R_0 within the standard final-size equation to calculate the proportion of exposed individuals at the end of a single epidemic peak, which allowed to the calculation the final attack rate based on R_0.[2]

All biological parameters related to transmission properties and course of infection were assumed to be the same for all locations, whereas parameters relating to life expectancy, population size, vector density (the free component of R_0) and seeding time (t_0) were estimated and assumed to be location specific.

Human life expectancy and population size were assumed to be known and fixed based on official statistics.[4–7] We assumed a fixed mosquito lifespan of 5 days, and fixed other model parameters such that the generation time of ZIKV was assumed to be ~20 days in line with previously published analyses on Zika transmission.[8] A sensitivity analysis was run where mosquito lifespan was fixed at 7 days, but this did not have a significant impact on the inferred microcephaly risk profile, although we note that R_0 estimates are conditional on the assumed generation time. A table summarising the chosen model parameters and their sources can be found in Table S2.

2.2 Microcephaly risk model

The second component of the model described the risk of a fetus developing microcephaly given that the mother was infected in a particular week during pregnancy. Fitting this risk profile as a curve rather than a set of per-trimester risk estimates captures more information regarding the width and shape of the gestational-time-varying risk profile at the resolution of weeks or days rather than per trimester. We used a scaled gamma distribution to characterise the shape and scale of this curve with only 3 free parameters - the shape, scale, and an additional scaling constant to increase the magnitude of the curve. This additional scaling constant was required as the sum under the risk profile did not need to sum to 1 as in the unmodified gamma distribution. The probability of developing microcephaly given infection was described as:

$$P'_m(x) = \frac{c}{\Gamma(x)\theta^k} x^{k-1} e^{-\frac{x}{\theta}} \tag{7}$$

Where $P'_m(x)$ is the probability of developing microcephaly given infection in gestational week x (0 to 39, where 0 is the first week of pregnancy); c is an additional scaling constant; θ is the gamma scale parameter; and k is the gamma shape parameter. The gamma distribution was chosen due to the flexible shape of the curve defined by a small number of parameters. Note that θ and k can be trivially manipulated to give the mean, mode and variance of the gamma curve. The gamma distribution, Γ was defined as:

$$\Gamma(x) = \int_0^\infty t^{x-1} e^{-t} dt \tag{8}$$

2.3 Combined model

Based on the transmission model and microcephaly risk model, the expected proportion of microcephaly affected births (Figure 1E) was calculated by multiplying these two components together. The probability of ZIKV-associated microcephaly affected birth at time, t, was therefore given by:

$$P_m(t) = \sum_{i=t-40}^{t} P_I(i) P'_m(i - t + 40) \tag{9}$$

Where $P_m(t)$ is the per live birth probability of a ZIKV-associated microcephaly birth at time t, $P_I(i)$ is the probability of an individual becoming infected at time i (and not before), and $P'_m(i - t + 40)$ is the probability of fetus developing microcephaly given ZIKV infection at gestational week $i - t + 40$. Essentially, the probability of a live birth being affected by ZIKV-associated microcephaly is the sum of all of the opportunities that the mother could have been infected and the fetus subsequently developed microcephaly in each of the 40 weeks of pregnancy preceding the birth.

Including a baseline microcephaly rate (ie. not associated with ZIKV) gives the probability of observing any microcephaly case at time t as:

$$P_{micro}(t) = \phi_{m,i}(1 - (1 - P_m(t))(1 - P_b)) \tag{10}$$

Where P_b is the baseline per birth microcephaly incidence rate and $\phi_{m,i}$ is the proportion of true cases that were reported in location i (less than one indicates underreporting, greater than one indicates overreporting). Multiplying this proportion by the total number of live births at time t, $B(t)$, gives the expected number of observed microcephaly-affected births at time t.

3 Data

3.1 Microcephaly and ZIKV incidence data

We searched the literature and Brazilian state health authority websites for reports of suspected ZIKV incidence and microcephaly cases in 2015 and early 2016, as described in the main text. To recap: we searched www.paho.org, www.who.int, Brazilian state-level ministry of health websites (eg. www.suvisa.ba.gov.br), and PubMed for the terms “zika” and “microcephaly”.

ZIKV and microcephaly incidence data from 2015 were available from publications and epidemiological reports for the states of Pernambuco, Rio Grande do Norte and Bahia (at state level and for the city of Salvador), though no useable data sets from 2015 were found for any other state. Monthly microcephaly incidence and births by state was also found online from the SINASC/CGIAE/SVS/MS system as reported previously.[9,10] An additional source of ZIKV incidence for all Brazilian states was also obtained from a publication in 2016;[11] however, the timing of the epidemic peak in these data suggested that incidence peaked in July 2015, contrasting with state-level reports which suggested an earlier peak. We also considered preliminary and later confirmed data ZIKV and microcephaly incidence published from the Brazilian ministry of health, which suggested a later ZIKV infection peak time compared to early state-level reports.[12,13] Microcephaly and ZIKV incidence data were obtained for Colombia at the national level.[14,15] Finally, we found confirmed microcephaly case

data for some locations (Rio Grande do Norte, Pernambuco, Northeast Brazil aggregated) and also confirmed ZIKV infection incidence for Colombia. Confirmed case reports were available for Colombia from weekly epidemiological bulletins; however, these did not include the date of report of confirmed cases, and we were therefore unable to extract incidence from reported cumulative cases.[16]

A summary of data included in the analyses can be found in Table S1. Some data sources were only available in graphical form, and these numbers were therefore extracted using a web digitizer (https://automeris.io/WebPlotDigitizer/). The results presented in the main text used:

1. Aggregated weekly confirmed microcephaly and notified ZIKV infection in pregnant women incidence data from Northeast Brazil;
2. Weekly confirmed/notified ZIKV infection and notified microcephaly incidence data from Colombia;
3. State-reported weekly notified microcephaly and ZIKV incidence from Bahia, Brazil;
4. State-reported confirmed weekly microcephaly incidence from Pernambuco, Brazil;
5. State-reported monthly confirmed/notified microcephaly and notified ZIKV incidence from Rio Grande do Norte;
6. Reported acute exanthematous illness (AEI) and notified microcephaly incidence from the city of Salvador, Bahia, Brazil (see below).

Model fitting using other data sources for the same locations, as well as fitting a single microcephaly risk profile to data from multiple locations simultaneously, was carried out but results are not presented here. Different data sources for the same location produced qualitatively similar risk profile estimates in terms of the window and timing of risk.

Numbers of live births were obtained for Brazil from the SINASC/CGIAE/SVS/MS system.[9,10] For Colombia, live births were obtained from a publication of microcephaly and ZIKV incidence in Colombia and ratified against country wide statistics.[15,17] Where reporting of live births was incomplete, we estimated the number of live births by averaging the number of births in the previous two years for the same dates. Where birth data was only available at a lower time resolution than reported incidence data, we assumed that the total number of births for that year were uniformly distributed across each day.

Extraction of incidence data from [13] was slightly more involved, as exact microcephaly case numbers were not provided. However, we were able to estimate the case count data given the methodology in this publication. [13] reported confirmed microcephaly incidence per 10,000 births and notified ZIKV infection incidence per 10,000 pregnant women, as well as the total number of microcephaly cases and monthly number of infected pregnant women. We could therefore infer the number of monthly births as follows:

$$\begin{aligned}
&\text{pregnant women(t)} = (\text{births(t)} * 9) + (\text{births} * 0.2) * 1.5 \\
&\text{pregnant women(t)} = \text{births(t)} * 9.3 \\
&\text{births(t)} = \text{pregnant women(t)}/9.3
\end{aligned} \tag{11}$$

Where pregnant women(t) is the number of pregnant women in month t (which is known) and births(t) is the number of births in month t. We then infer the number of microcephaly cases per month as:

$$\text{microcephaly cases(t)} = \text{births(t)} * \text{microceph incidence(t)} \tag{12}$$

Where $\text{microcephaly cases(t)}$ is the number of microcephaly cases reported in month t and $\text{microceph incidence(t)}$ is the per birth incidence of microcephaly in month t.

Note that we use the total population size for Northeast Brazil as the denominator for per capita incidence, as the reporting rate parameter (described below) accounts for the fact that infected pregnant women only represent a fraction of the true infected population.

All data are available to download in human readable form in the accompanying R package here. The files `data_sources.csv` and `data_key.csv` describe the sources of these data and the column names respectively. Note that `startDay` and `endDay` refer to the first and last day that the reporting period covers; `buckets` refers to the number of days that that reporting window covers; and all dates were converted to integers (where 1 day = 1), and 01/01/2013 was taken as day 0.

3.2 Model parameters

Model parameters related to ZIKV transmission were obtained from the literature as described in Table S2. Parameters were predominately chosen based on a previously published transmission model, with point values chosen to give a generation time of approximately 20 days.[8] Values used were as described in Table S6 of [8], with the intrinsic latency period here taken as the intrinsic incubation period described in [8] less 1.5 days to reflect the assumption that infectiousness starts 1.5 days before symptom onset. Given a fixed generation time, the shape of the SEIR model predicted incidence curve was allowed to vary depending on the value of R_0. As R_0 is comprised of multiple correlated parameters, all components of R_0 other than the vector density per human were fixed. Vital statistics (human life expectancy and population size) for particular locations are described in Table S3.

4 Model fitting

Using the expected number of observed microcephaly cases as described above, we fit the model to available microcephaly incidence and live birth data to estimate model parameters. The likelihood function and fitting algorithm are described below.

4.1 Microcephaly incidence likelihood

The log likelihood of observing a time series of microcephaly cases for a given location i was given by:

$$l(D_i|\Psi, \theta_i) = \sum_t \log P(d_t|\Psi, \theta_i) \tag{13}$$

Where D_i is the observed number of microcephaly cases over time; $D_i = \{d_t\}_{t=1}^{T}$; d_t is the number of microcephaly cases observed at time t; θ_i is the set of location-specific parameters (eg. mosquito density, N_H) and Ψ is the set of universal model parameters that apply to all locations (eg. p_{MH}, α_H). Observed microcephaly incidence was assumed to be binomially distributed such that:

$$P(d_t) \sim B(n = n(t), p = P_{micro}(t)) \tag{14}$$

Where $n(t)$ is the total number of births observed at time t which was known; and $P_{micro}(t)$ is the proportion of microcephaly affected births at time t as defined by the model parameters Ψ and θ_i; and B is the binomial probability mass function.

4.2 ZIKV incidence and combined likelihood

Note that the reporting rate scaling parameter, $\phi_{m,i}$ was assumed to be location-specific as described above. Note also that this log likelihood is easily extended to incorporate ZIKV incidence data as well as microcephaly incidence data $I_i = \{i_t\}_{t=1}^{T}$ as:

$$l(D_i, I_i|\Psi, \theta_i) = \omega \sum_{t_m} \log P(d_t|\Psi, \theta_i) + (1 - \omega) \sum_{t_i} \log P(i_t|\Psi, \theta_i) \tag{15}$$

Where I_i is the set of ZIKV incidence data for location i; θ_i is the set of location-specific model parameters; Ψ is the vector of universal model parameters; and ω is an optional weighting parameter that scales the contribution of the ZIKV incidence data to the likelihood. t_m and t_i indicate that microcephaly and ZIKV incidence data do not necessarily cover the same observation period (as ZIKV incidence would predate microcephaly incidence).

The binomial likelihood of observing a ZIKV case at a given time, t is given by:

$$P(i_t) \sim B(n = N, p = \phi_{I,i} P_I(t)) \tag{16}$$

Where i_t is the observed ZIKV incidence at time t; N is the total population size; $\phi_{I,i}$ is the state-specific proportion of true ZIKV cases that observed incidence represents (through under or overreporting, or misdiagnosis) and $P_I(t)$ is the model predicted probability of becoming infected at time t as described above.

All results in the main text are based on independent fits to data from single locations, giving parameter estimates unique to each location. However, for completeness, we show the complete log likelihood function combining information from multiple locations:

$$l(D|\Psi, \theta) = \sum_{n=i} l(D_i, I_i|\Psi, \theta_i) \tag{17}$$

Where $D = \{D_i\}_{i=1}^{n}$; D_i is the microcephaly incidence data from location i; $I = \{I_i\}_{i=1}^{n}$; I_i is the ZIKV incidence data from location i; θ_i is the set of parameters specific to location i; $\theta = (\theta_1, \theta_2, ..., \theta_n)$ is the vector of all location-specific parameters; Ψ is the set of universal model parameters; and n is the number of locations included in the analysis.

Using the above log likelihood, we defined the log posterior probability function to be:

$$\log \pi(\Psi, \theta|D, I) = \log p(\Psi, \theta) + \sum_{n=i} l_i(D_i, I_i|\Psi, \theta_i) \tag{18}$$

Where $p(\Psi, \theta)$ is the prior probability of the universal and location-specific model parameters; and $\pi(\Psi, \theta|D, I)$ is the posterior probability. Note that the prior probability sits outside of the summation. We assumed uniform priors for all free model parameters with upper and lower bounds described in Table S2.

4.3 Model fitting without ZIKV incidence data

We were able to fit the model to microcephaly incidence data alone by setting the weighting of the ZIKV incidence component, ω, of the log likelihood to 0. However, some knowledge of the timing of the ZIKV epidemic peak was available for some locations based on reported incidence. In these analyses, we incorporated information on the timing of ZIKV epidemic peak to help constrain the timing of peak infection risk. This peak time can be considered a function of the SEIR model parameters (ie. the peak of ZIKV incidence generated by the SEIR model). This was used to inform a uniform prior distribution as follows:

$$p(t_{peak}) \sim unif(a - \frac{b}{2}, a + \frac{b}{2}) \tag{19}$$

Where t_{peak} is the model generated ZIKV peak incidence time in that location; a is the peak time of the ZIKV epidemic in that location based on the day of maximum reported ZIVK incidence; and b is the width of the uniform window around this peak time, representing uncertainty in the timing of the peak. Here, we chose b to be 120 days to represent a ~4 month window of uncertainty around the timing of the ZIKV epidemic peak. Parameter values that give $t_{peak} < a - \frac{b}{2}$ or $t_{peak} > a + \frac{b}{2}$ are therefore assigned a probability of 0. a was 06/05/2015 for Bahia; 03/02/2016 for Colombia; 16/03/2015 for Pernambuco; and 13/05/2015 for Rio Grande do Norte based on the sources shown in Table S1.

4.4 MCMC algorithm

Using the equation above, we defined a binomial likelihood of observing a number of microcephaly cases at any unit time given a set of model parameters and known number of births. We defined the likelihood function such that we could calculate the combined likelihood of observing ZIKV and microcephaly incidence data from any number of geographical locations conditional on both universal and location-specific parameters. Furthermore, we incorporated the potential for location specific over and underreporting through reporting rate parameters, ϕ_I and ϕ_m. Total births were either known from the data, or estimated by taking the average of the previous two years' births in the same time period. Where weekly birth data was required from monthly data, we evenly spread the monthly number of births across the number of days in that month and then summed the number of births for each 7 day block.

Using the log posterior function defined above, we fit the model to available incidence data using an MCMC framework written in R and C++ (`lazymcmc`) with the `rlsoda` package. Chains were run for 2000000 iterations with a 750000 iteration burn in and adaptive period. The chains were run to ensure that a sufficient effective sample size was achieved for all model parameters or at least 200, with convergence assessed using the Gelman-Rubin diagnostic tool with the coda package in R. The result of this analysis was posterior distribution estimates for all free model parameters conditional on the included data.

5 Forecasting the second wave of microcephaly incidence

We added four additional model parameters to quantify potential changes in behaviour and reporting rates across two seasons of microcephaly and ZIKV incidence that would explain the two seasons of observed data, where only one wave of microcephaly incidence was observed despite two waves of ZIKV incidence. We tested the following hypotheses:

1. Microcephaly reporting accuracy was different before the most recent change in microcephaly case definition on 13/03/2016 for cases reported through the Registro de Eventos em Saúde Pública (RESP) database in Brazil. Under/over-reporting of microcephaly may therefore have been different before and after this date.

2. Following the WHO announcement of a Public Health Emergency of International (PHEIC) concern on 01/02/2016, women may have begun to seek targeted abortions for microcephaly affected pregnancies <24 weeks of gestation, which would manifest itself as a probability of seeking an abortion given development of ZIKV-associated microcephaly in a particular week of pregnancy.
3. The proportion of ZIKV-affected births after 11/11/2015 may have decreased relative to reported per capita ZIKV incidence, either through pregnant women taking additional precautions to avoid infection relative to the rest of the population or through delaying pregnancy entirely.
4. ZIKV incidence reporting accuracy may have changed after 11/11/2015 after the WHO/PAHO issued an alert accompanied by improved laboratory detection guidelines for ZIKV. Under/over-reporting of ZIKV infection may therefore have been different before and after this date.

We define the probability of not developing microcephaly during the first $i-t+40$ days of pregnancy as $\bar{bp_i} : (1-bp)^{i-t+40}$. We also make the following notational simplification to give the probability of a fetus developing ZIKV-associated microcephaly at time i, $P_i : P_I(i)P'_m(i-t+40)$. The expected proportion of observed microcephaly affected live-births was therefore given by:

$$P_{micro}(t) = \phi_m \sum_{i=t-40}^{t} \begin{cases} \bar{bp_i}(P_i + bpP_i + bp) & t < t_{switch} \\ (1-a_r)\bar{bp_i}((1-b_r)P_i + bp(1-b_r)P_i + bp) & t \geq t_{switch} \;\&\; (i-t+40) < t_{abortion} \\ \bar{bp_i}((1-b_r)P_i + bp(1-b_r)P_i + bp) & t \geq t_{switch} \;\&\; (i-t+40) \geq t_{abortion} \end{cases} \quad (20)$$

Where $P_I(i)$ is the probability of becoming infected at time i; $P'_m(i-t+40)$ is the probability of developing microcephaly given infection in gestational week $i-t+40$; t_{switch} is the time at which behavioural changes could have occurred (assumed to be 01/02/2016, mechanism 3); b_r is the proportion of potentially affected births that were avoided (mechanism 3); a_r is the proportion of microcephaly-affected births that were aborted (mechanism 2); and $t_{abortion}$ is the gestational time before which abortions could occur, assumed to be 24 weeks (mechanism 2); bp is the baseline daily *probability* of developing microcephaly during pregnancy (note that this is different to the previous definition of baseline microcephaly, P_b, which was defined as a rate per observed live birth rather than a probability per day during pregnancy).

Overall, this term gives the probability of an individual not developing baseline microcephaly during the first $i-t+40$ days of pregnancy and either developing ZIKV-associated microcephaly, baseline microcephaly or both on day i. This term is then multiplied by the probability of observing that microcephaly case (ie. the reporting rate), given by ϕ_m, which was assumed to have one value before and one after 13/03/2016 (mechanism 1).

As our model did not explicitly include seasonality, we used reported ZIKV incidence directly to estimate the per capita infection risk across two seasons. We calculated the probability of becoming infected with ZIKV as:

$$P_I(t) = \begin{cases} \frac{I(t)}{\phi_{inc_1}} & t < t_1 \\ \\ \frac{I(t)}{\phi_{inc_2}} & t \geq t_1 \end{cases} \tag{21}$$

Where $I(t)$ is the observed per capita ZIKV incidence at time t; ϕ_{inc_1} is the proportion of true ZIKV cases that were reported before t_1; ϕ_{inc_2} is the proportion of true ZIKV cases that were reported after t_1; and t_1 is the time at which reporting behaviour was assumed to have changed, fixed here at 11/11/2015.

Although we did not use the SEIR model generated force of infection to predict infection risk in this analysis, we did include the SEIR model to estimate the proportion of ZIKV cases that were reported in the first wave. In other words, the SEIR-model predicted incidence curve was fit to reported ZIKV incidence for the single wave only as a component of the likelihood function. The other component of the likelihood was the probability of observing the two seasons of microcephaly-affected births given $P_{micro}(t)$. If ZIKV transmission followed SEIR-like dynamics with a mosquito vector, then the shape of the incidence curve would give inferential power regarding the relationship between observed and true incidence dynamics, allowing us to estimate plausible values of t_0, R_0 and ϕ_{inc_1} that might generate observed incidence data for that season. Values for ϕ_{inc_2} were therefore estimated relative to the inferred value of ϕ_{inc_1}

Finally, the number of aborted births could be calculated by estimating the proportion of microcephaly-affected pregnancies that were aborted ($P_{micro}(t)$ defined above, ignoring the impact of reporting rate), divided by the proportion of microcephaly-affected pregnancies that weren't aborted ($P_{micro}(t)$ defined above, but replacing $1 - a_r$ with a_r, ignoring the impact of reporting rate) multiplied by the observed number of microcephaly cases.

5.1 Parameter estimation

Parameter estimation was performed using the same likelihood function and MCMC framework described above. Where the joint impact of all of the four mechanisms was estimated, all of the parameters relating to these mechanisms were assumed to be unknown. Where the contribution of each mechanism alone was estimated, we fixed the contribution of all but the one relevant mechanism parameter to be 0 and refit the model to the incidence data for Bahia, Brazil.

6 Fitting to data from Salvador, Brazil

We obtained reported acute exanthematous illness (AEI) attributed to Zika virus and microcephaly incidence in Salvador, Brazil during 2015.[18] We assumed that reported AEI was proportional to the true incidence of ZIKV during this time, and scaled the weekly reported incidence to give a final attack rate in line with seroprevalence estimates for Salvador. Scaling was done by dividing reported AEI cases each week by ϕ_I which was calculated as follows:

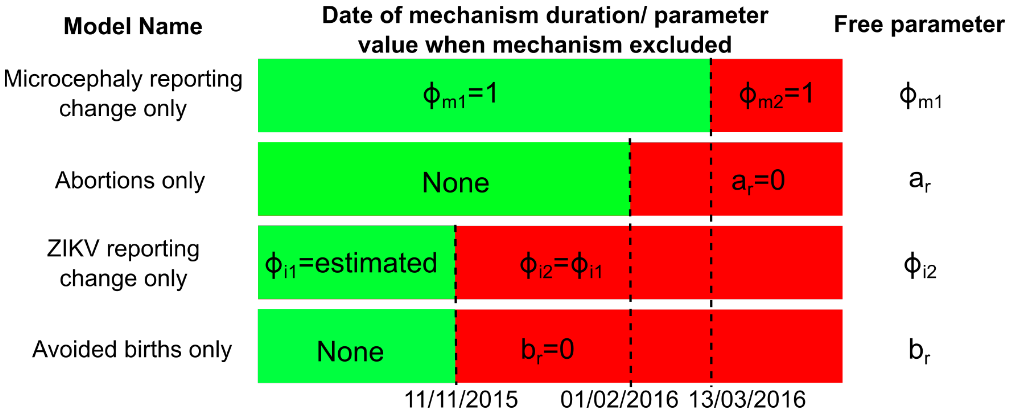

Figure 2: **Schematic of forecasting analysis mechanism switch times.** Left hand column describes the model variant name corresponding to this mechanism as used in Table S5. The middle column demonstrates the time at which reporting or behaviour may have switched (green before the switch, red after). The values shown in the middle column show the assumed values or contributions of these parameters when the mechanism is excluded. The Right hand column shows the parameter that is estimated as a free parameter for that analysis. For example, if microcephaly reporting is assumed to stay the same, then $\phi_{m1} = \phi_{m2} = 1$ for all dates. If microcephaly reporting change is the mechanism under consideration, then ϕ_{m1} is estimated as a free model parameter. In the joint impact analysis, all parameters in the "free parameter" column are estimated.

$$\phi_I = \frac{\sum_t I(t)/N}{AR} \tag{22}$$

Where $I(t)$ is the reported incidence at week t, N is the population size of Salvador, and AR is the reported attack rate based on seroprevalence. We placed a uniform prior on AR such that the total attack rate was between 59.4 and 66.8%.[19] N was inferred by dividing the total number of reported AEI cases by the reported total incidence per 1000 persons (giving 2922037); life expectancy was assumed to be the same as Bahia overall at 73.1 years. We calculated weekly number of live births by backtracking from the reported microcephaly incidence in this time period and the total number of microcephaly cases reported. Paploski et al. report "367 newborns with suspected microcephaly (15.6 cases/1,000 newborns during July 2015–February 2016, which peaked at 31.4 cases/1,000 newborns in December)", suggesting that there were 23,526 newborns in this period. We assumed that these births were distributed uniformly across each week such that there were 420 births per week from July 2015 – February 2016. The microcephaly reporting rate, ϕ_m, was assumed to be 100%.

Model fitting was then carried out as above; fixing all model parameters other than ϕ_I; α; β; and c as described in Table S2. Note that in this analysis the SEIR model component is not included.

References

1. MacDonald G. The Epidemiology and Control of Malaria. 1957.

2. Diekmann O, Heesterbeek J. Mathematical Epidemiology of Infectious Diseases: Model Building, Analysis and Interpretation. John Wiley & Sons; 2000.

3. Keeling M, Danon L. Mathematical modelling of infectious diseases. Br Med Bull. 2009;92: 33–42.

4. Brazil life expectancy [Internet]. Available: ftp://ftp.ibge.gov.br/Tabuas_Completas_de_Mortalidade/Tabuas_Completas_de_Mortalidade_2014/notastecnicas.pdf

5. Brazil population size [Internet]. Available: ftp://ftp.ibge.gov.br/Estimativas_de_Populacao/Estimativas_2015/serie_2001_2015_TCU.pdf

6. Colombia life expectancy [Internet]. Available: https://www.dane.gov.co/files/investigaciones/poblacion/series_proyecciones/proyecc3.xls

7. Colombia population size [Internet]. Available: http://www.worldometers.info/world-population/colombia-population/

8. Ferguson NM, Cucunubá ZM, Dorigatti I, Nedjati-Gilani GL, Donnelly CA, Basáñez M-G, et al. Countering Zika in Latin America. Science. 2016;387: e41918–e41918. doi:10.1126/science.aag0219

9. Johansson MA, Mier-y-Teran-Romero L, Reefhuis J, Gilboa SM, Hills SL. Zika and the Risk of Microcephaly. N Engl J Med. 2016;375: 1–4. doi:10.1056/NEJMp1605367

10. Microcefalia no brazil – 2000 a 2016 [Internet]. Available: https://public.tableau.com/profile/bruno.zoca#!/vizhome/Painel2-microcefalia/Painel2-Microcefalia

11. Faria NR, Azevedo R do S da S, Kraemer MUG, Souza R, Cunha MS, Hill SC, et al. Zika virus in the Americas: Early epidemiological and genetic findings. Science. 2016;352: 345–349. doi:10.1126/science.aaf5036

12. Oliveira WK de, Carmo EH, Henriques CM, Coelho G, Vazquez E, Cortez-Escalante J, et al. Zika Virus Infection and Associated Neurologic Disorders in Brazil. N Engl J Med. 2017;376: 1591–1593. doi:10.1056/NEJMc1608612

13. Oliveira WK de, França GVA de, Carmo EH, Duncan BB, de Souza Kuchenbecker R, Schmidt MI. Infection-related microcephaly after the 2015 and 2016 Zika virus outbreaks in Brazil: a surveillance-based analysis. Lancet. 2017;390: 861–870. doi:10.1016/S0140-6736(17)31368-5

14. PAHO/WHO. Pan American Health Organization / World Health Organization. Zika - Epidemiological Report Colombia. September 2017. Washington, D.C. [Internet]. 2017. Available: http://www.paho.org/hq/index.php?option=com_docman&task=doc_view&gid=35139&Itemid=270&lang=en

15. Cuevas EL, Tong VT, Rozo N, Valencia D, Pacheco O, Gilboa SM, et al. Preliminary Report of Microcephaly Potentially Associated with Zika Virus Infection During Pregnancy — Colombia, January–

November 2016. MMWR Morb Mortal Wkly Rep. 2016;65: 1409–1413. doi:10.15585/mmwr.mm6549e1

16. 2017 boletín epidemiológico semana 52 [Internet]. Available: https://www.ins.gov.co/buscador-eventos/Paginas/Vista-Boletin-Epidemilogico.aspx

17. Nacimientos por área de ocurrencia y sexo, según grupos de edad de la madre, total nacional - Cifras con corte a 7 de diciembre de 2017 (publicadas el 22 de diciembre de 2017) [Internet]. Available: https://www.dane.gov.co/index.php/estadisticas-por-tema/salud/nacimientos-y-defunciones/nacimientos

18. Paploski IA, Prates APP, Cardoso CW, Kikuti M, Silva MMO, Waller LA, et al. Time Lags between Exanthematous Illness Attributed to Zika Virus, Guillain-Barré Syndrome, and Microcephaly, Salvador, Brazil. Emerg Infect Dis. 2016;22: 1438–1444. doi:10.3201/eid2208.160496

19. Netto EM, Moreira-Soto A, Pedroso C, Höser C, Funk S, Kucharski AJ, et al. High Zika Virus Seroprevalence in Salvador, Northeastern Brazil Limits the Potential for Further Outbreaks. MBio. 2017;8: e01390–17. doi:10.1128/mBio.01390-17
